# Circular RNAs—The Road Less Traveled

**DOI:** 10.3389/fmolb.2019.00146

**Published:** 2020-01-10

**Authors:** Ashirbad Guria, Priyanka Sharma, Sankar Natesan, Gopal Pandi

**Affiliations:** ^1^Department of Plant Biotechnology, School of Biotechnology, Madurai Kamaraj University, Madurai, India; ^2^Department of Genetic Engineering, School of Biotechnology, Madurai Kamaraj University, Madurai, India

**Keywords:** circRNA, biogenesis, long non-coding RNA, miRNA sponging, backsplicing

## Abstract

Circular RNAs are the most recent addition in the non-coding RNA family, which has started to gain recognition after a decade of obscurity. The first couple of reports that emerged at the beginning of this decade and the amount of evidence that has accumulated thereafter has, however, encouraged RNA researchers to navigate further in the quest for the exploration of circular RNAs. The joining of 5′ and 3′ ends of RNA molecules through backsplicing forms circular RNAs during co-transcriptional or post-transcriptional processes. These molecules are capable of effectively sponging microRNAs, thereby regulating the cellular processes, as evidenced by numerous animal and plant systems. Preliminary studies have shown that circular RNA has an imperative role in transcriptional regulation and protein translation, and it also has significant therapeutic potential. The high stability of circular RNA is rendered by its closed ends; they are nevertheless prone to degradation by circulating endonucleases in serum or exosomes or by microRNA-mediated cleavage due to their high complementarity. However, the identification of circular RNAs involves diverse methodologies and the delineation of its possible role and mechanism in the regulation of cellular and molecular architecture has provided a new direction for the continuous research into circular RNA. In this review, we discuss the possible mechanism of circular RNA biogenesis, its structure, properties, degradation, and the growing amount of evidence regarding the detection methods and its role in animal and plant systems.

## Introduction

Circular RNAs (CircRNAs) have recently spread into the non-coding RNA world. The circRNAs are formed by the covalent circularization of a 3′ downstream donor and the 5′ upstream acceptor in an alternate form of pre-mRNA splicing by a process called backsplicing (Szabo and Salzman, 2016). However, the mechanisms of biogenesis, nuclear export, degradation, and the functional significance of circRNAs, remain unclear or exist as proposed theories. Mounting evidence on the presence of circRNAs in all the organisms tested so far shows that the circRNAs are an integral part of living systems (Salzman et al., 2012, 2013; Memczak et al., 2013; Zhang et al., 2013; Zhang X.-O. et al., 2016; Zhang Y. et al., 2016; Ashwal-Fluss et al., 2014; Starke et al., 2015; Pamudurti et al., 2017; Tan et al., 2017; Yang et al., 2017). Despite this, our understanding of their structural and functional aspects is limited. In this review, we have made an attempt to highlight the promising discoveries that have been made in the field of circRNAs in the recent past.

## History

The first circRNA ever seen by an electron microscope was a plant viroid, and it was subsequently proven to be so due to its circular nature and through the use of various biochemical analyses, such as analyzing its resistance to degradation by snake venom phosphodiesterase and 5′-phosphorylation (Sanger et al., 1976). Similarly, the first ever animal virus reported to contain circRNA was the Hepatitis delta virus (HDV) (Kos et al., 1986). However, the animal kingdom contributed immensely to the understanding of a plethora of diverse avenues within circRNA biology. The 1990s witnessed a few endogenous circRNAs originating from deleted in colorectal carcinoma (DCC) (Nigro et al., 1991), Sex-determining region Y (SRY) (Capel et al., 1993), proto-oncogene ETS-1 (Cocquerelle et al., 1992), Cytochrome P450 2C24 (Zaphiropoulos, 1996, 1997), and Sodium/Calcium exchanger (NCX1) (Li and Lytton, 1999) genes in humans, mice, rats, and monkeys. These were discovered by sequencing the PCR products containing the backsplice junctions with the 5′ exon present downstream of the 3′ exon (Jeck and Sharpless, 2014). Similarly, in the beginning of the 21st century, a muscle blind (MBL) gene from *Drosophila* (Houseley et al., 2006), human antisense non-coding RNA in the INK4 locus (ANRIL) (Burd et al., 2010), and cerebellar degeneration-related autoantigen 1 antisense (CDR1as) (Hansen et al., 2011) genes were reported to express circRNAs. These findings suggested that the formation of circRNAs was an odd and irregular phenomenon during splicing, and this was termed to be a mis-splicing process (Cocquerelle et al., 1993).

The concept of mis-splicing on the formation of circRNAs was changed entirely when thousands of circRNAs were reported to exist independently of different human and mouse cell lines (Salzman et al., 2012; Jeck et al., 2013; Memczak et al., 2013). Since then, circRNAs have been discovered in a wide range of organisms, including Zebrafish (Shen et al., 2017; Liu H. et al., 2019; Sharma et al., 2019), *Caenorhabditis elegans* (Cortés-López et al., 2018), *Saccharamyces cerevisiae, Schizosaccararomyces pombe, Plasmodium falciparum*, and *Dictyostelium discoideum*. Similarly, the presence of circRNAs was also reportedly found in *Arabidopsis thaliana* (Wang P. L et al., 2014), *Oryza sativa* ssp. *Japonica, Oryza sativa* ssp. *Indica, Nicotiana benthamiana* (Guria et al., 2019), and in 12 other plant species (Chu et al., 2017). However, their identification and characterization reveal that the biogenesis of circRNAs originates through the act of backsplicing and not through mis-splicing, as was reported previously. This growing amount of evidence suggests circRNAs have an important role in the eukaryotic tree of life (Wang P. L et al., 2014).

## Biogenesis

**Figure 1:**
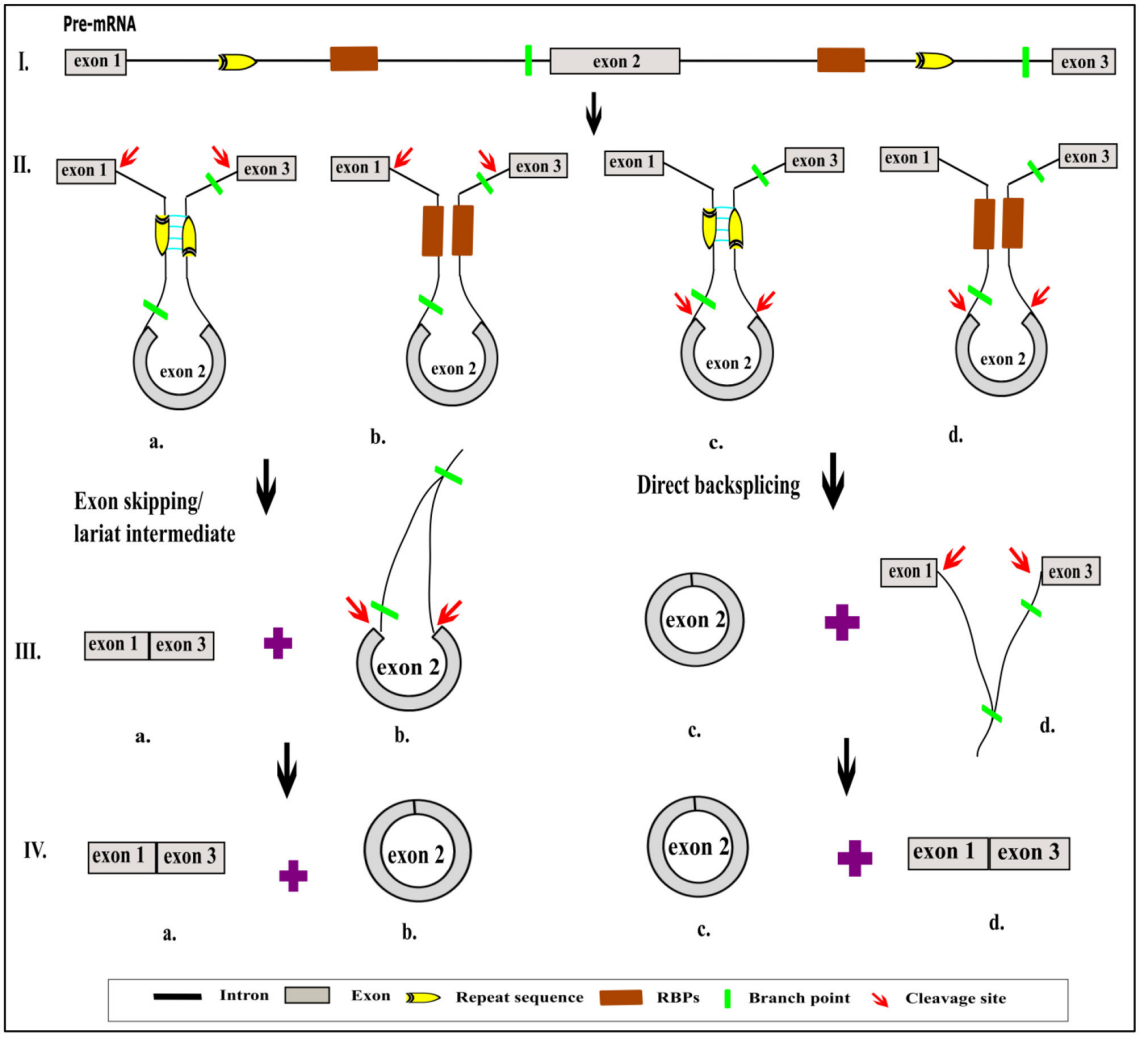
Schematic representation of role of *cis* sequence and *trans* factor in different models of circRNA biogenesis. Normally, the pre-mRNA is enriched with the exonic and intronic region (I). The non-coding intronic region harbors the highly conserved sequence in the 5′ and 3′, which is essential for splicing by spliceosomal machinery (I). In addition to the conserved sequence, flanking introns consist of a repeat sequence or RBP site, which help to bring the 5′ and 3′ ends of intervening exon closer together due to either the base pairing (IIa, IIc) or by the binding of RBP (IIb, IId). Due to this proximity, circRNAs are generated by exon skipping or direct backsplicing (IIIa, IVa). Exon skipping produces linear RNA first, followed by the circularization of an intervening exon along with the formation of a lariat containing flanking introns (IIIb). Subsequent splicing yields a circular exonic RNA (IVb). In contrast, in direct backsplicing, exonic circRNA is generated first (IIIc, IVc) and is then followed by an exon–intron lariat (IIId). The latter is processed further to convert it into linear RNA (IVd). The pictorial representation is not to scale.

Numerous circRNAs were serendipitously discovered from scrambled exons in different human cell types (Salzman et al., 2012). These reports have suggested that circRNA may significantly contribute to exon scrambling, but all scrambled exons need not necessarily be circRNAs. The two most pivotal models of circRNA biogenesis are by direct backsplicing and exon skipping or by lariat intermediate formation (Chen and Yang, 2015) (Figure 1). Both models give rise to circRNAs and linear RNAs from the flanking regions, which raises further questions regarding the frequency of occurrence of one model over another. The exon-skipped linear RNA is either degraded (Egecioglu et al., 2012; Bitton et al., 2015) or results in a truncated protein that is different from the native protein.

**Figure 2:**
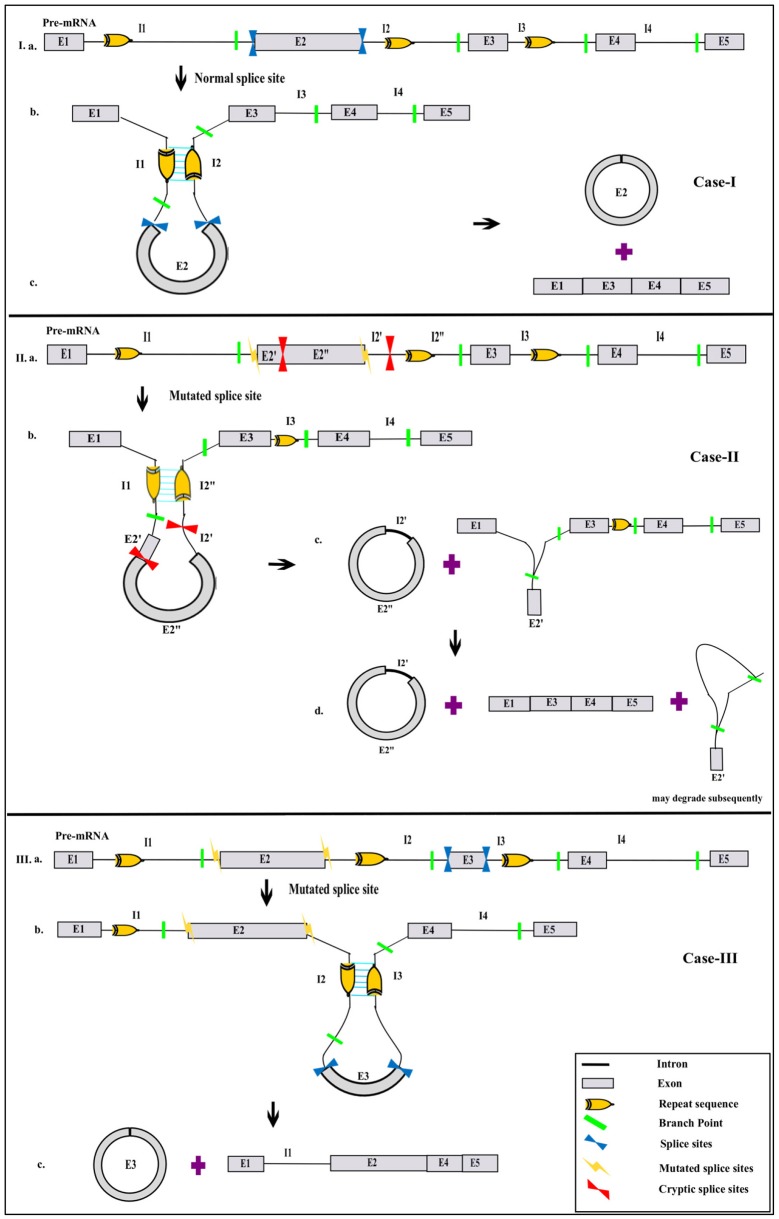
Possibilities of circRNA biogenesis during splice site mutation. Canonical splice sites in exon(s) of pre-mRNA (Ia) surrounded by a repeat sequence in flanking introns brings the 5′ and 3′ ends of intervening exons closer (Ib), thereby resulting in the formation of a linear RNA and a circRNA (Ic). However, mutation in the natural splice site of pre-mRNA (IIa, IIIa) allows the spliceosome to access the next cryptic splice site possible, present within either an exon or intron (IIb). Cleavage at the cryptic site results in production of circRNA with a shortened exon and an extended intron before the cryptic site, whereas the leftover exon may remain, along with the exon–intron lariat (IIc). Subsequent splicing of the latter leads to the formation of an exon-skipped linear RNA, followed by the possible degradation of a remnant exon (IId). In another scenario of natural splice-site mutation where the immediate cryptic sites are unavailable, the spliceosome may be more inclined toward the next possible splice site of the exon that is flanked by a repeat sequence (IIIb). This may result in the generation of a new exonic circRNA and an exon-skipped linear RNA with intron retention (IIIc). The pictorial representation is not to scale.

Recent studies have led to the discovery of many essential *cis* and *trans* factors that have a positive or negative regulatory effect on circRNA biogenesis (Figure 1). CircRNA production requires the joint involvement of spliceosomal machinery and the natural splice sites (Starke et al., 2015) through a co-transcriptional mechanism (Ashwal-Fluss et al., 2014; Huang and Shan, 2015). Hence, competition might occur between the canonical splicing and backsplicing mechanisms in the same sequence to form linear mRNA or circRNA, respectively (Ashwal-Fluss et al., 2014; Chen and Yang, 2015). The presence of roughly 1% of circRNAs among mRNAs reveals that canonical splicing is more prominent than backsplicing (Salzman et al., 2013). However, post-transcriptional regulation of circRNA biogenesis is also reported in Fused in Sarcoma (FUS) gene-depleted motor neurons *in-vitro* (Errichelli et al., 2017). Mutations in natural splice sites from 5′GU to 5′CA decreases circRNA production (Ashwal-Fluss et al., 2014). *In-vitro* studies using single exon minigenes show that, when both the 5′ and 3′ splice sites are mutated, the spliceosomal machinery is inclined toward the next cryptic splice site, which leads to an increase or decrease in the circumference of the circle (Figure 2). It may ultimately result in weakening of the circularization efficiency. On the other hand, it has also been validated that any sequence can be circularized if the last three nucleotides in the 5′ and 3′ spliceosomal recognition sites remain unchanged (Starke et al., 2015). Conversely, most of the plant circRNAs are joined by non-canonical splice sites (Ye et al., 2017; Chu et al., 2018a,b; Guria et al., 2019); the probable reason for this could be the flexibility in binding of the spliceosome machinery. Due to high complementarity, the microRNA (miRNA)-mediated cleavage of circRNAs is possibly another striking reason for the lower number of circRNAs in plants, as shown in *Vitis vinifera* L. (Gao et al., 2019). Moreover, the identification of miRNA binding and cleavage sites in circRNA, either by rapid amplification of cDNA ends (RACE) or degradome sequencing, is difficult due to lack of a 5′ cap and 3′ poly-A tail. This is compelling evidence, and there might yet be other unidentified mechanisms involved in the biogenesis of circRNA in plants (Chu et al., 2018a,b). Overall, the biogenesis of circRNA is regulated by spliceosomes and the recognition of both the canonical and non-canonical splice junctions. This eventually results in the biogenesis of different types of circRNAs, such as exon–intron circRNA, exon-intergenic circRNA, etc. (Table 1), which are all backspliced from different genomic regions.

**Table 1:** Types of circRNAs.

**CircRNA types**	**Abbreviation**	**1st Splice site on**	**2nd splice site on**	**Remarks on splice sites**
Exonic circRNA	e-circRNA	Exon	exon	It can be on the same or multiple exons
Intronic circRNA	i-circRNA	Intron	intron	Both sites on the single intron
Exon–intron circRNA	ei-circRNA	exon	Intron	1st splice site on exon and 2nd splice site on intron, may or may not be spanned by the intervening exon or/and intron.
Intron–exon circRNA	ie-circRNA	Intron	Intron	On different introns spanned by one or multiple exons
UTR circRNA	u-circRNA	UTR	UTR	Both can be on 5′ or 3′ or there can be one at 5′ and one on 3′
UTR-exon circRNA	ue-circRNA	UTR	Exon	circRNA formed at 5′ or 3′ end
UTR-intron circRNA	ui-circRNA	UTR	Intron	circRNA formed at 5′ or 3′ end with single or multiple exons in between
Intergenic circRNA	ig-circRNA	Intergenic	Intergenic	Both sites on same intergenic region
Intergenic-genic circRNA	igg-circRNA	Intergenic	Genic	Genic could be from exon or intron
Across genic circRNA	Ag-circRNA	Genic	genic	Sites will be spanned by intergenic region

The role of *cis*-flanking sequences cannot be avoided in the circularization of intervening sequence(s). Flanking introns or exons are found to be much longer (~3 fold) in circularizing the intervening exonic or intronic sequences, respectively, than the normal corresponding sequences, which generally undergo linear splicing (Jeck et al., 2013; Barrett and Salzman, 2016; Bolha et al., 2017). Indeed, the presence of complementary, reverse complementary, direct repeats (DR), and inverted repeats (IR) indicates that they are enriched in longer flanking introns, which bring the 5′ and 3′ sequences close enough for circularization. The presence of Alu and IRAlu repeats in the ZWILCH locus yields circRNA formation in mice but not in humans, thereby implying that circRNA expression is species specific in nature (Hansen et al., 2011). In plants, however, the miniature inverted-repeat transposable elements (MITES) found in flanking introns of rice exonic circRNAs (Lu et al., 2015), and reverse complementary pairs of LINE1-like elements (LLECRPs) in *Zea mays* (Chen et al., 2018) play a critical role in circRNA biogenesis. Understanding the role of repetitive sequences in flanking regions of circRNAs in polyploid species will be of great interest to the assessment of genome complexity (Chu et al., 2018b). This is because the repetitive or reverse complementary sequences in longer flanking introns cover only for a limited amount of exonic circRNA production in plants (Ye et al., 2015; Chu et al., 2018a). However, Starke et al. (2015) proved that reverse complementary repeats (RCR) of 103 nucleotides (nt) caused a 1.3-fold increase in the efficiency of circularization, suggesting that the presence of longer flanking introns is not always mandatory. To prove the above observation, a minimum of 30–40 nt RCR in the flanking sequence has been shown to be enough for circularization (Liang and Wilusz, 2014; Ivanov et al., 2015). The length of single exon has also been found to be longer to produce single exonic circRNA than normal exons, which undergo canonical splicing (Bolha et al., 2017). Biogenesis of circular intronic RNAs (ciRNA) requires a motif having 7 nt GU-rich element near the 5′ splice site and 11 C-rich nucleotides closer to the branch point. The presence of these elements may help to escape from cleavage (2′-5′ linkage) by debranching enzyme thereby favoring the circRNA biogenesis (Zhang et al., 2013).

Apart from the *cis* sequences, the *trans* factors, such as the RNA-binding proteins (RBPs) are found to be important for circularization of exonic circRNAs, which have a conserved binding site in the flanking introns. Excess Muscleblind-like splicing regulator 1 (MBNL1) proteins bind to the conserved motifs present in the flanking introns of the second exon of their own gene and regulate circMBNL1 biogenesis (Ashwal-Fluss et al., 2014). The RBP Quaking (QKI), an alternative splicing factor, binds to the intronic conserved motifs to form circRNA during human epithelial–mesenchymal transition (EMT). QKI also has the ability to circularize any linear RNA provided its ends are connected to quaking binding sites (Conn et al., 2015). Similarly, FUS controls the regulation of circRNA biogenesis in mouse motor neurons by binding to ~1,500 nt long flanking introns conserved in human pluoripotent stem cell-derived motor neurons (Errichelli et al., 2017). Apart from the cytoplasmic exonic circRNAs, nuclear exonic circRNAs were also reported (Jeck and Sharpless, 2014; Errichelli et al., 2017), which reveals a new direction toward finding novel functions of these nuclear circRNAs. *C. elegans* are not rich in repeat sequences but contain reverse complementary sequences. Owing to this, Adenosine (A) to Inosine (I) editing by adenosine deaminase acting on RNA1 (ADAR1) is frequent in the intronic sequences, which are responsible for circularizing intervening exons (Ivanov et al., 2015). The knocking down of ADAR1 and ADAR2 in human and mouse cell lines and in *Drosophila* increases circRNA expression without perturbing the linear RNA transcript (Ivanov et al., 2015; Rybak-Wolf et al., 2015). Thus, the biogenesis of circRNAs is regulated by divergent pathways, and understanding this detailed mechanism would require further research.

## Properties

### Stability

One of the most attractive features of circRNA is its stability. The circular nature of 2′-5′ linked or 5′-3′ backspliced RNA confers its existence for more than 48 h (Jeck and Sharpless, 2014), as evidenced by its resistance to exonuclease degradation when compared to linear RNA (half-life <10 h). This could be one of the major reasons behind its detection, even if it constitutes only 1% of poly-A RNA (Salzman et al., 2013). However, the stability of circRNAs in serum is around 15 s, and the probable reason behind this could be the presence of circulating endonucleases (Jeck and Sharpless, 2014).

### Conserved Nature

The compilation of various RNA-seq data sets so far show the conserved nature of circRNA across different species in both animals and plants; for example, circRNA originating from genes like Imprinted in Prader-Willi syndrome (IPW), Plasmacytoma Variant Translocation 1 (PVT1) (Salzman et al., 2013), Homeodomain Interacting Protein Kinase 2 (HIPK2), HIPK3, and KIAA0182 (Jeck et al., 2013) are reported to be present in both mice and humans. Similarly, the expression of circMBNL1 was reported in human and *Drosophila* heads (Ashwal-Fluss et al., 2014). More than 700 common exonic circRNAs, shared between *O. sativa* and *A. thaliana* (Ye et al., 2015), were reported, and a similar observation was also observed between *O. sativa Indica* and other plants (Guria et al., 2019). These examples depict the conservation of circRNA that originated from the genomic locus, which evolved with a paralogous or orthologous nature.

### Expression Specificity

The expression of circRNA is tissue specific (Memczak et al., 2013; Salzman et al., 2013; Gao et al., 2015; Zhao W. et al., 2017), isoform specific, and development specific, as seen in *O. sativa, A. thaliana* (Ye et al., 2015, Gao et al., 2015), and in WI-38 fibroblast cells (Panda et al., 2016). The differential expression of circbHLH93 has been shown in eight developmental stages of *Phyllostachys edulis* (Wang Y. et al., 2019). Stress-specific circRNA expression was also reported in *O. sativa* during phosphate imbalance (Ye et al., 2015), drought stress in *T. aestivum* (Wang Y. et al., 2017), and cold tolerance in *V. vinifera* (Gao et al., 2019). However, experimental validations are yet to be established mechanistically. Tissue/cell-specific circRNA expression has been shown. CircSRY, for example, is expressed in adult mouse testes (Capel et al., 1993) but present as linear *Sry* mRNA in the developing genital ridge (Barrett and Salzman, 2016). CircZFAND6 was absent, and linear *Zfand6* was present in the NHLF cell line. However, A549 cells expressed a single circular isoform of ZFAND6, whereas dual circular isoforms are found in other cell lines. Longer circAMBRA1 were highly expressed in MCF-7 cells, whereas a higher expression of short circAMBRA1 was observed in HepG2 cells (Salzman et al., 2013). The competitive edge of canonical splicing over backsplicing yields more linear RNA than circRNA, and the opposite is also found to be true. For example, circCAMSAP1 expression was 20 times more abundant than its linear counterpart in many of cell lines tested. Overexpression of circRNA, as compared to its linear counterpart, was also observed in 50 other genes examined in A549, AG04450, and HeLa cell lines (Salzman et al., 2013). Similarly, circRNA coming from the KIAA0182 gene locus in the human fibroblast (Hs68) cell line expressed a 10-fold increase over its linear mRNA (Jeck et al., 2013). Recently, we have also reported that ~20% of circRNAs are highly expressed than linear RNAs, which are spliced out from the same locus in *O. sativa* ssp. *Indica* (Guria et al., 2019).

### Types

CircRNAs are classified based on the location of the splice junction in the genome from which they originate. The three basic types of circRNAs are exonic, intronic, and exonic–intronic. Chu et al. (2018a), however, have recently summarized 10 different types of circRNA (Table 1).

Previously, the presence of antisense circRNA, overlapping circRNA, and sense overlapping circRNA was also reported in *T. aestivum* (Wang Y. et al., 2017). Since then, the types of circRNAs have been accumulating; International Nomenclature is strictly required to maintain uniformity and to avoid confusion in the identification of the circRNAs by the global circRNA research community.

### CircRNA–RBP Interaction

Emerging evidence has proven that circRNA–RBP interaction is fundamental to various dimensions of cellular processes. The interaction of RBP with circRNA may cause the RBP to be sequestered away from its action or circRNA to act as an RBP sponge. HuR, a translational activator, binds to PABPN1 mRNA and enhances its translation. Recently, HuR has been found to interact with circPABPN1, curtailing HuR binding with PABPN1 mRNA and curtailing its translation—a classic example of an RBP getting sponged by a circRNA (Abdelmohsen et al., 2017). Similarly, circFOXO3 sequesters cell cycle proteins, such as CDK2 and p21, and it reduces their interaction with cyclin A and cyclin E, resulting in G1- or S-phase cell cycle arrest (Du et al., 2016). Likewise, an excess MBL protein can bind and help to circularize a portion of its own pre-mRNA, resulting in the decreased production of its cognate mRNA (Ashwal-Fluss et al., 2014). CircRNA can also act as a positive regulator. CircPAIP2, circEIF3, and circANKRD52 bind to Pol II transcription machinery of their corresponding genes, and they augment the expression of linear transcript (Zhang et al., 2013). To further strengthen the circRNA–RBP interaction process, Circinteractome has been found to analyze the presence of such networks in humans. The pipeline has identified 117,000 circRNA that interact with EIF4A3. Similarly, it also detects hsa_circ_0024707 harboring 85 AGO2 binding sites and is thus called an RBP super sponge (Dudekula et al., 2016).

### Structure

**Figure 3:**
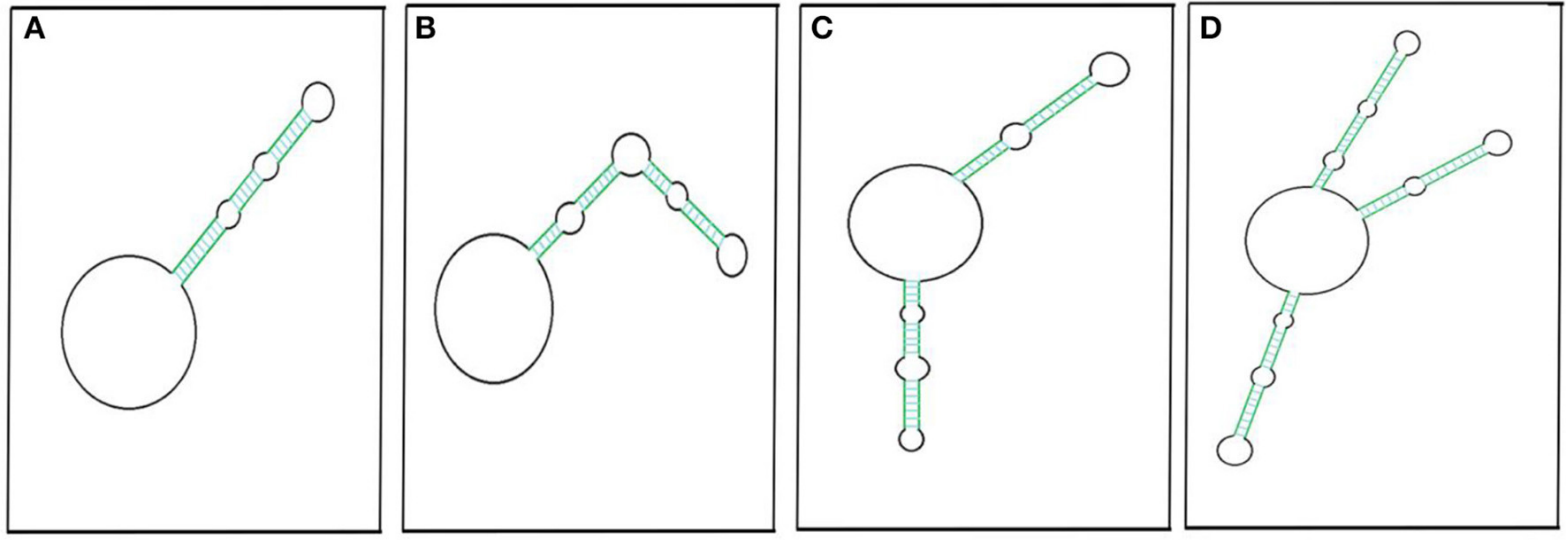
Pictorial representation of circRNAs with putative secondary imperfect duplex(es). CircRNA with a double-stranded RNA (dsRNA) foldback structure can be made possible by having single **(A,B)**, double **(C)**, or multiple projections **(D)**. Colored parallel lines represent different sizes of complementary regions (dsRNA regions), and black loops represent different sizes of non-complementary regions. The pictorial representation is not to scale.

An RNA molecule that is circular, is without any free ends, and contains an ejected arm comprising complementary regions capable of forming a double strand (Figure 3) can also be called a circRNA (Liu C.-X et al., 2019). The circRNA first discovered, the potato spindle tuber viroid (PSTVd), is monomeric and contains rod-shaped structures with multiple distinct loops (López-Carrasco and Flores, 2017). The structure is stable with minimum free energy in the absence of any protein interaction, leaving PSTVd as a naked form (López-Carrasco and Flores, 2017).

Liu C.-X et al. (2019) have likewise reported 26 circRNAs from multiple cell lines that have been found to have ≥1 intra-molecular dsRNA duplex secondary structure. Most of these structures are 16–26 bp long, though there are two exceptions: circDHX34 has one 29 bp-long dsRNA projection and circPPP1CB has two projections, of which one is 32 bp long. These intra dsRNA duplexes are reported to have an inhibitory function. The dsRNA binding protein PKR masks the phosphorylation sites and inhibits its activation, highlighting the functional importance of the secondary structure within circRNA. Besides this, these duplexes almost resemble the dsRNA foldback loops, which have shown to be imperative for miRNA biogenesis (Czech and Hannon, 2010; Kumar et al., 2017). The endoribonuclease Dicer may recognize similar secondary structures in circRNAs for miRNA biogenesis. However, the possible mechanism of miRNA generation from circRNAs is yet to be determined. Recently, we reported extremely lengthy circRNAs from *O. sativa* ssp. *Indica* that encompass sequences for different miRNAs (Guria et al., 2019). Hence, the processivity of these mature miRNAs may be linked to circRNA sequences. Along with this, internal modification, such as N6-methyladenosine (m6A) of an miRNA precursor (Alarcón et al., 2015), which is also frequent in circRNAs (Zhou C. et al., 2017), may facilitate miRNA processing. Based on this evidence, we hypothesized the possibility of miRNA biogenesis resulting from circRNAs.

Furthermore, a DNA–RNA hybrid loop, or R-loop, from exon 6-skipped circRNA of *SEP3* has been identified in *A. thaliana*. This hybrid loop decreased its own transcription elongation, favoring enhanced exon skipping and leading to a phenotypically defective SEP3.3 variant (Conn et al., 2017). On the other hand, the non-naturally occurring exon 5-skipped circRNA, having a high secondary complementary intramolecular structure, had a reduced probability of R-loop formation which was increased upon heat denaturation. This further highlights how, if exon 6-skipped circRNA had a secondary complementary structure, the probability of R-loop formation would have been inadequate to produce the SEP3.3-generated phenotype (Conn et al., 2017). Thus, the role of the secondary structure of circRNA emphasized the transcriptional control of its own and, possibly, other target genes with high complementarity. In addition, temperature might play a role in the formation of circRNA variants, as shown in the case of *SEP3*. It has been hypothesized that circRNA generation is favored under low temperature condition, resulting in more variants of circRNA (Conn et al., 2017). Due to the prevalence of frequent alternative backsplicing, circRNA isoforms with varied internal sequence compositions are expected. Based on this, Gao et al. (2016) designed CIRI_AS using forward splice junction (FSJ) reads and backward splice junction (BSJ) reads to explore the internal structures of circRNAs, such as intramolecular dsRNA loops (Liu C.-X et al., 2019).

### Localization

Several reports have confirmed the presence of exonic circRNA in cytoplasm (Jeck et al., 2013; Memczak et al., 2013; Jeck and Sharpless, 2014), whereas the intron-retained circRNAs, like exon–intron circRNA and intronic circRNA, are found exclusively in the nucleus (Zhang et al., 2013; Barrett and Salzman, 2016; Ebbesen et al., 2017). Similarly, it is believed that circRNAs containing retained introns, like intron–intergenic circRNA, may be present in the nucleus. Surprisingly, in the Neuro2a (N2a) cell line, it has been shown that the exonic circRNAs are also localized in the nucleus (Errichelli et al., 2017). However, the nuclear export mechanism of exonic circRNAs remains unknown. One possibility could be the escape of circRNAs when the nuclear envelope disintegrates during mitosis (Jeck and Sharpless, 2014). Recently, Huang et al. (2018) correlated the export mechanism of circRNAs in relation to its length. The *Drosophila* nuclear export protein Hel25E and its human homolog UAP56 (DDX39B) have a conserved K-K/S-L-N motif that is responsible for the export of long circRNA (>1,200 nt) to the cytoplasm through the nuclear pore complex. Similarly, another member of the human exportin family, URH49 (DDX39A) is accountable for the export of <400 nt short circRNA into the cytoplasm. URH49 is dependent on the R-S-F-S motif, but swapping with the K-K/S-L-N motif in the URH49 results in the alteration of its property and it begins to behave like Hel25E/UAP56. However, the nuclear export protein involved in short circRNA export in *Drosophila* is yet to be deciphered. Moreover, it has been hypothesized that long and short circRNAs may follow the NXF1–NXT1 and PHAX–CRM1 pathway, respectively, for cytoplasmic export; this is similar to mRNA, and this needs to be determined experimentally (Huang et al., 2018). Another study has highlighted the nuclear export of circRNA by the direct binding of IGF2BP1 to circRNA followed by exportin2 (XPO2) attachment in an RAN-GTP-dependent manner (Ngo et al., 2019, 24th RNA Society Annual Meet, Krakow).

## Methods of Detection and Validation

As mentioned above, sporadic evidence concerning the presence of circRNA before the end of the millennium hypothesized it as being a result of splicing noise. However, reports on the identification of circRNAs in the current decade (Salzman et al., 2012, 2013, Memczak et al., 2013) and other studies thereafter have led researchers to develop methodologies to characterize the circRNAs. Numerous computational tools have been developed that have been further improvised to detect circRNAs by setting out various criteria that are specific to certain organisms, species, or genera from the high-quality sequencing reads. Indeed, software designed exclusively for a particular organism can also be used to check for the presence of circRNA in another organism by modifying the default parameters. A DCC pipeline developed for the identification of circRNAs from the heart (Jakobi et al., 2016), for example, is used to detect circRNAs from plants like *O. sativa* and *N. benthamiana* (Guria et al., 2019). Some of the well-known and frequently used computational pipelines for circRNA detection are CIRI (Gao et al., 2015), Circexplorer (Zhang et al., 2014), Circexplorer2 (Zhang X.-O. et al., 2016), circRNA_finder (Westholm et al., 2014), KNIFE (Szabo et al., 2015), Mapsplice (Wang et al., 2010), DCC (Cheng et al., 2016), CIRI_AS (Gao et al., 2016), Segemehl (Hoffmann et al., 2014), circseq_cup (Ye et al., 2017), and pCircRNA_finder (Chen L. et al., 2016), which check the presence of at least one backsplice event in NGS data. Of course, they all have their own advantages and limitations. As a result, genome-wide identification of circRNAs from any organism may actually under-represent the total number of circRNAs detected, and this is also accompanied by missing out on the abysmally expressed circRNAs (Szabo and Salzman, 2016). The pcircRNA_finder is, so far, the only pipeline designed for circRNA determination in plants by combining multiple software to detect backsplicing reads (Chu et al., 2018a,b). This calls for simultaneous analysis using different software to confirm the extent of similarity and number of novel circRNAs. Various animal and plant circRNA databases are being created where new additions are recorded as and when they are reported. Some of the non-exhaustive plant circRNA databases include PlantcircBase (Chu et al., 2017), which has a repository of more than 115,000 circRNAs from 16 different plants. AtcircDB, meanwhile, is meant exclusively for *Arabidopsis* circRNA (Ye et al., 2019). The PlantCircNet (Zhang P. et al., 2017) is meant for visualizing the plant circRNA-miRNA–mRNA interaction networks. Similarly, CircFunBase is the animal circRNA database and is a repository of functional circRNAs (Meng et al., 2019). CircR2Disease (Fan et al., 2018) is designed for the circRNAs involved in various diseases, and circBase (GlaŽar et al., 2014) is designed for circRNAs reported from humans, *Drosophila*, mice, and *C. elegans*. Some of the above tools can be used for the comparative expression of linear RNAs and circRNAs by calculating the read counts for each type of RNA and comparing them with total read counts. Nevertheless, experimental validation is a must in order to rule out the false positives generated from NGS analysis.

The inherent circular nature provides resistance to degradation from exoribonucleases, such as RNase R, augmenting the enrichment of circRNAs before subjecting them to NGS (Suzuki et al., 2006; Vincent and Deutscher, 2006). Treatment with RNase R is more appropriate as it will likely decrease the detection of a backsplice junction generated from template switching, trans-linear splicing, or a genome duplication event (Barrett and Salzman, 2016). Later, divergent RT-PCR/qRT-PCR and subsequent Sanger sequencing and/or northern hybridization can be performed to validate the accurate identification of the NGS-derived circRNAs (Hansen et al., 2011; Wang Z. et al., 2017; Cortés-López et al., 2018; Guria et al., 2019).

Small nuclear RNAs (snRNAs) or the highly structured double-stranded RNA, having 3′-overhangs that are shorter than 7 nt, are resistant to digestion by RNase R (Suzuki et al., 2006; Vincent and Deutscher, 2006; Pandey et al., 2019). In order to enhance the enrichment of pure circRNAs from a pool of total RNA, Poly-A tailing followed by poly-A depletion can be carried out on the leftover complex-structured linear RNA after RNase R treatment. This will categorically reduce the remaining linear RNA that is otherwise present after RNase R treatment. The efficiency of digestion can be examined by PCR for the absence/presence of linear RNA (Pandey et al., 2019).

A gel trap assay, where RNase R usage can be omitted, is yet another method using low-melting agarose that is heated and mixed with total RNA prior to loading in agarose gel. This causes the circular molecules to be trapped inside the well, which can be extracted, purified, and deep sequenced for the presence of circRNA (Jeck and Sharpless, 2014; Barrett and Salzman, 2016). Similarly, total RNA can also be run on vertical non-denaturing two-dimensional polyacrylamide gel electrophoresis (2D-PAGE) in two different directions perpendicular to each other. Electrophoretic migration of circular molecules is slow and shows an arch-like pattern, as compared to linear molecules, due to its trapping in the cross-linked gel (Jeck and Sharpless, 2014). The entire arch can be gel eluted and deep sequenced for global circRNA identification. In yet another method, use of RNase H, which can cleave a RNA–DNA hybrid, can also be employed for circRNA identification by assigning a short DNA probe that is complementary to a region of circRNA. A single nick at the bound region will make it linearized for circRNA, or two different bands if it is linear RNA when run on an agarose gel (Jeck and Sharpless, 2014; Barrett and Salzman, 2016).

Backspliced events from total RNA can also be detected by designing a circRNA ampli-seq panel consisting of known circRNAs and their corresponding linear counterparts followed by NGS (Zaghlool et al., 2018). Thus, it has the advantage of simultaneous quantification of circRNA and its corresponding linear RNA in a specific condition. In yet another strategy, fluorescently labeled padlock probes containing the complementary region of the backspliced junction of a circRNA were designed, followed by rolling circle amplification (RCA). The amplified product was detected by epifluorescence or could be sequenced for particular circRNA identification. However, in both the cases, the sequence of the circRNA has to be known to either design the ampli-seq panel or padlock probes.

Recently, we employed a new technique where we exploited the properties of multiple displacement amplification (MDA) to harness even low-expressed circRNAs from *O. sativa* ssp. *Indica* and *N. benthamiana* for the first time (Guria et al., 2019). The MDA products were digested, cloned, and sequenced to check for the presence of junction sites by comparing the plant circRNA database. Additionally, the MDA products were deep sequenced at much lesser reads and analyzed for genome-wide circRNA identification, which resulted in 1,875 and 9,242 circRNAs from *O. sativa* ssp. *Indica* and *N. benthamiana*, respectively. This method proves to be much cheaper than the traditional RNA-seq method and can be applied on any unannotated genome organism.

## Alternative Circularization

**Figure 4:**
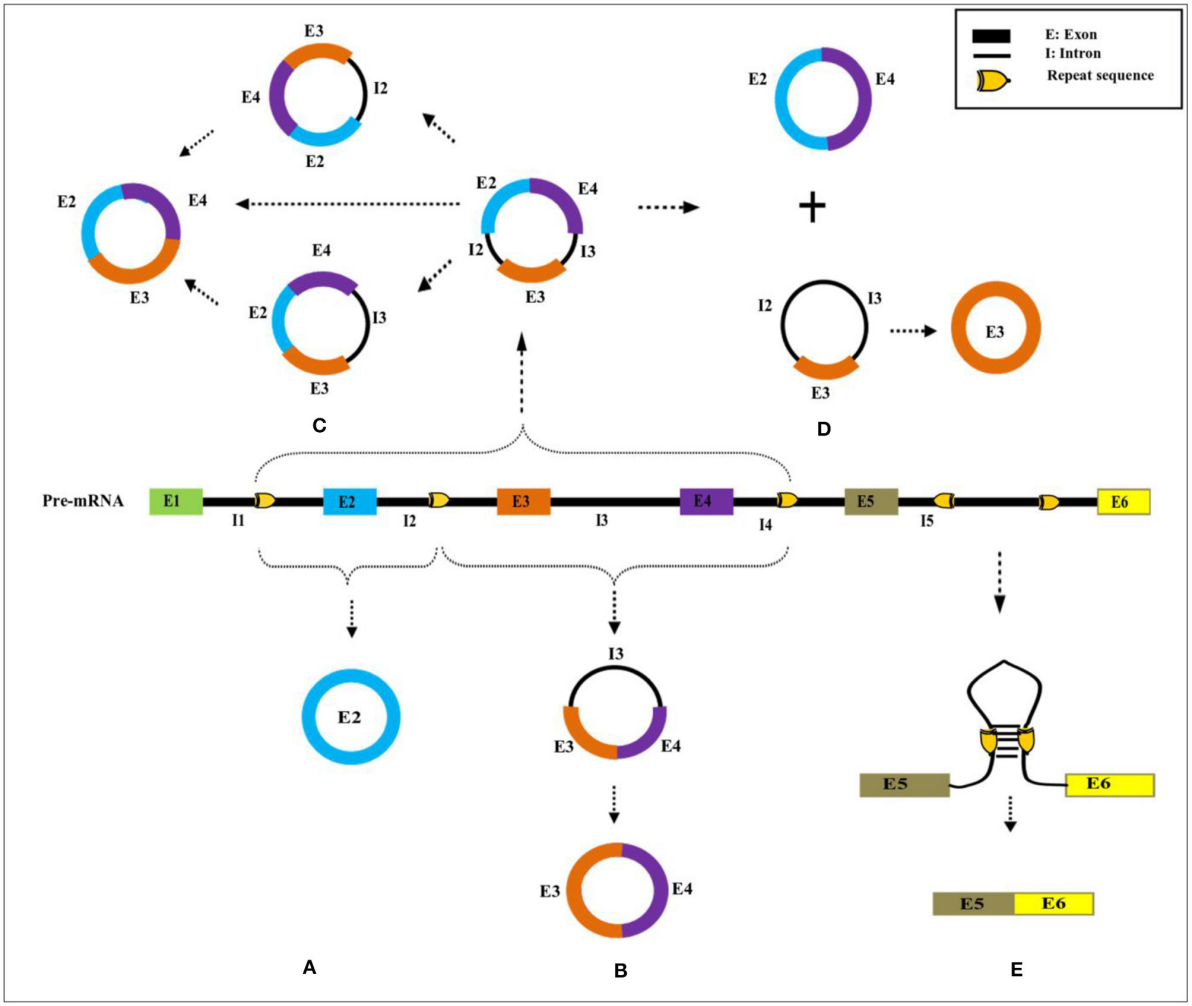
Possible alternate backsplicing and canonical splicing. Presence of *cis* sequences across introns in pre-mRNA results in formation of circRNAs of varying sizes that are comprised of different exons with or without introns **(A–D)**. Presence of indirect repeat sequence in the same intron causes sequential joining of flanking exons to form linear RNA **(E)**. The pictorial representation does not reflect in actual scale.

Based on the computational prediction, it has been determined that a specific gene locus could yield more than one circRNA of different lengths via alternative backsplicing. This mechanism is primarily attributed to association with the competition of putative RNA pairs across introns, which favor the circularization of exons (Zhang et al., 2014; Chen and Yang, 2015; Zhang X.-O. et al., 2016). For instance, an IR sequence present in >2 introns, as shown in Figure 4, can result in multiple numbers of varying sizes of circRNAs, either exonic or exonic–intronic circRNA (EiciRNA). But what determines the dominance of one intronic pair over the other possibly forming pairs in a generation of circRNAs is yet to be determined. On the other hand, the presence of the same IR within a single intron results in canonical linear splicing, which then yields the sequential joining of two flanking exons (Chen and Yang, 2015) (Figure 4). Subsequently, EiciRNAs (Figure 4) can undergo another round of backsplicing reaction to generate only exonic circRNA and the skipping of intervening introns, which might undergo degradation by spliceosomal factors (Chen and Yang, 2015).

Two different types of alternative backsplicing can be possible: (a) alternative 5′ backsplicing and (b) alternative 3′ backsplicing (Zhang X.-O. et al., 2016). The presence and usage of more non-canonical backsplice junctions anywhere in the genome renders an increase in alternative circularization efficiency, which yields different types of circRNAs. The splice variants of circRNA with the same junction sites can be detected by a circRNA-RCA approach, which further provides an opportunity to identify the full-length circRNAs (Das et al., 2019). However, prior knowledge of circRNA sequence information is a must to design primers. Indeed, most of the plant circRNAs are isoforms of varying lengths from the same locus (Guria et al., 2019). Similarly, human *Camsap1* is shown to produce 7 exonic circRNA isoforms in H9 cells (Zhang et al., 2014). Large numbers of circRNA isoforms are generated from a gene having a longer transcript (Khan et al., 2016; Guria et al., 2019). For example, the human cardiac-specific *Titin* gene with 365 exons has been shown to result in 80 different circRNAs. The ryanodine receptor (*RyRs*) gene with 107 exons has been reported with around 59 RyR-specific circRNAs (Khan et al., 2016). Likewise, the S-adenyltransfer gene can produce two circRNAs that have different backsplicing sites and where both share an overlapping parental region in *P. edulis* (Wang Y. et al., 2019). However, the expression of circRNA isoforms will vary depending on the changes in physiological conditions. Thus, alternative circularization is a major criterion for circRNA diversity within a genome (Zhang X.-O. et al., 2016), and the generation of the genome is again correlated with biotic and abiotic factors.

## CircRNA-Derived Pseudogenes

Pseudogenes from linear RNA, which are known for retrotransposition, are abundantly present in mammalian genomes, whereas they are significantly less so in plants (Prade et al., 2018). In an analogy with the former observation, a parallel concept was correlated with circRNA-derived pseudogenes (Dong et al., 2016). CircRNA can undergo reverse transcription containing a backsplice junction that can integrate randomly at multiple positions of the genome. This could be either with the complete or partial pseudogene sequence but must contain the backsplicing site, thereby altering the genome architecture. For example, 33 high-confidence pseudogenes were found to be generated from circRFWD2 containing the exon 6 and exon 2 as backsplicing sites in mice. However, the same are not found in rats or other primates as determined using a circpseudo computational pipeline, suggesting the possible occurrence of divergent evolution (Dong et al., 2016). On the other hand, a circSATB1-derived pseudogene could be found in both mice and rats, explaining the possible occurrence of evolutionarily conserved retrotransposition. Conversely, circPRKDC- and circCAMSAP1-derived pseudogenes could be found in gorillas and chimpanzees but not in rhesus monkeys. This elucidates how retrotransposition might have occurred very recently during the course of evolution. Sometimes, a pseudogene may be found in one mouse strain but not in another, which explains the strain-specific retrotransposition within the species (e.g., circDIAP3-derived pseudogene). Pseudogenes derived from circRNA generally possess few Adenosines (A) at their 3′end as compared to linear RNA-derived pseudogenes. However, the mechanism of integration remains unknown (Dong et al., 2016). Nevertheless, it may be possible that new circRNA(s) might emerge from the pseudogene-integrated locus in the genome. Thus, a generation of circRNA followed by the retrotransposition of pseudogenes generated from circRNA is a dynamic process. The formation of new circRNA(s) from the newly integrated site, which may again be retro transposed, thereby may impart in the development of evolution. However, evidences for the same are lacking probably because a balance is being maintained by small RNAs such as hc-siRNA/PIWI RNA to counteract the effect of retrotransposition (He X-J. et al., 2011).

## Functions

### MiRNA Sponging

Much evidence has emphasized the putative role of circRNAs in gene regulation. One of the proven functions of circRNA is the inhibition of the miRNA function by binding the target miRNAs directly or indirectly by a process commonly referred to as miRNA sponging. CiRS-7/circCDR1as is a classic example having 74 miR-7 binding sites in humans and at least 63 conserved binding sites across 32 vertebrates (Memczak et al., 2013; Li et al., 2018). Imperfect complementary between ciRS-7-miR-7 strongly suppresses the miR-7 availability to its mRNA targets (Hansen et al., 2013) involved in various cancers (Kefas et al., 2008; Reddy et al., 2008) (Table 2) and neuro-degenerative disorders such as Parkinson disease (Junn et al., 2009) and Alzheimer's disease (Lukiw, 2013). On the other hand, ciRS-7 has perfect complementarity with miR-671 between the 10–11 nt along with extended complementarity beyond the 12 nt; as a result of this, ciRS-7 is cleaved by Argonaute-2 (AGO-2) (Hansen et al., 2011). Similarly, circSRY sponges miR-138 by binding to at least 16 sites, and it thus possibly plays a role in the progression of cancer and Parkinson's disease (Hansen et al., 2013; Qu et al., 2017). A single circRNA can bind to a single or many miRNAs at one or more sites (Memczak et al., 2013; Guria et al., 2019) by either perfect or near-perfect binding. For example, circITCH can bind to miR-138, miR-17, and miR-124, whereas circFOXO3 can bind to miR-22, miR-136^*^, miR-138, miR-149^*^, miR-433, miR-762, miR-3614-5p, and miR-3622b-5p (Qu et al., 2017). However, only a small number of circRNAs were found to have a sponging effect (Chu et al., 2018a,b; Guria et al., 2019), which suggested the possibility of having simultaneous other putative functions. It is intriguing to note that all the circRNAs that display sponging activity are localized in the cytoplasm, and most of them are exonic circRNAs (Kulcheski et al., 2016).

**Table 2:** Functional characterization of circRNAs and their significance in disease curbing.

**Disorder type**	**Molecular feedback axis and their expression levels**	**CircRNA information**	**Remark(s)**	**Computational pipeline used**	**References**
Breast cancer (BC)	Hsa_circ_0004771 (high)/miR-653 (low)/ZEB2 (high)	13 binding sites for different miRNAs	*In vitro* and *In vivo* studies. ZEB2 protein executes EMT. MiR-653 suppresses *Zeb2* expression by binding at its 3′-UTR and acts as a tumor suppressor that is sponged by hsa_circ_0004771	Circinteractome, TargetScan	Xie et al., 2019
Lung adenocarcinoma (LUAD)	CircMTO1 (low)/miR-17 (high)/QKI-5 (low)	CircMTO1 derived from exon 2 and 3 of *Mto1* gene (318 bp)	*In vitro* and *In vivo* studies. Tumor suppressor CircMTO1 sponges miR-17, allowing for enhanced QKI-5 expression, which, in turn, retards proliferation by inactivating the Notch signaling pathway	RNAhybrid, miRanda, TargetScan	Zhang et al., 2019
Non-small cell lung carcinoma (NSCLC)	CircP4HB (high)/miR-133a-5p (low)/Vimentin (high)	Derived from exon of *P4hb* gene located on chromosome 17.	*In vitro* and *In vivo* studies. CircP4HB promotes EMT by sponging tumor suppressor miR-133a-5p.	Arraystar software	Wang T. et al., 2019
Bladder carcinoma (BCa)	CircDOCK1 (high)/hsa_miR_132-3p (low)/Sox5 (high)	CircDOCK1 has potential binding sites for five different miRNAs	*In vitro* and *In vivo* studies. Targeting circDOCK1 reduced cell viability and retarded migration of tumor	PicTar, TargetScan	Liu P. et al., 2019
	CircHIPK3 (low)/miR-558 (high)/HPSE (Heparanase) (high)	Derived from exon 2 of *Hipk3* gene (1099 bp)	*In vitro* and *In vivo* studies CircHIPK3 attenuates cell migration, invasion and angiogenesis in BCa by sponging miR-558 which down-regulates HSPE expression and its downsteam targets MMP-9 and VEGF	miRanda, PITA, RNAhybrid	Li et al., 2017
	CircTCF25 (high)/miR-103a-3p and miR-107 (low)/CDK6 (high)	Derived from exon(s) of *Tcf25* gene located on chromosome 16	*In vitro* and *In vivo* studies. miR-103a-3p and miR-107 negatively regulates CDK6, which, along with Cyclin D, controls G1 to S transition by inactivating RB1 via phosphorylation	miRanda, TargetScan, DIANA-miRPath, DAVID	Zhong et al., 2016
	Hsa_circ_0068871 (high)/miR-181a-5p (low)/FGFR3 (high)	Derived from exon 4–8 of *Fgfr3* gene	*In vitro* and *In vivo* studies Hsa_circ_0068871 sponges miR-181a-5p, which is a tumor suppressor and regulates FGFR3 expression. FGFR3 activates STAT3, which triggers tumor development	circBase, CircNet, CircInteractome, TargetScan, RNA 22v2	Mao et al., 2019
	CircITCH (low)/miR-17, miR-224 (high)/p21, PTEN (low)	Derived from several exons of *Itch* gene	*In vitro* and *In vivo* studies. CircITCH is a tumor suppressor molecule that can sponge miR-17 and miR-224, thereby downregulating p21 and PTEN involved in BCa cell proliferation	Starbase V2.0, Circinteractome	Yang C. et al., 2018
Hepatocellular carcinomas (HCC)	Cir_0005986 (low)/miR-129-5p (high)/Notch1 (low)	Derived from gene *Prdm2* (PR/SET Domain 2) localized on chromosome 1	*In vitro* studies Cir_0005986 affects proliferation via regulating G0/G1 to S phase transition	DIAN mirPath v.3, Arraystar software, miRTarBase	Fu et al., 2017
Laryngeal squamous cell carcinoma (LSCC)	CircRASSF2 (high)/miR-302b-3p (low)/IGF-1R (high)	Derived from *Rassf2* gene present on chromosome 20	*In vitro* and *In vivo* studies CircRASSF2 promotes tumorigenesis by upregulating IGF-1R, which is a target of miR-302b-3p. Overexpressed circRASSF2 secreted in serum by exosomes	TargetScan, PicTar, miRanda	Tian et al., 2019
Pancreatic ductal adenocarcinoma (PDAC)	CircRNA_100782 (high)/miR-124 (low)/IL-6, STAT3 (high)	Derived from sequence on chromosome 11	*In vitro* and *In vivo* studies CircRNA_100782 sponges miR-124, a negative regulator of proliferation in PDAC. miR-124 targets IL-6 and STAT3, which are crucial for cell growth and survival	TargetScan	Chen et al., 2017
Gastric cancer (GC)	CircRNA_100269 (low)/miR-630 (high)/LPHN2 (low)	Derived from exon of *Lphn2* gene present on chromosome 1.	*In vitro* studies miR-630 negatively regulates the circRNA_100269 and, thereby, downregulates the expression of its linear variant *Lphn2*	TargetScan, miRanda	Zhang Y. et al., 2017
Esophageal squamous cell carcinoma (ESCC)	CircITCH (low)/miR-7 (high)/ITCH (low)	Derived from exons 6-13 of *Itch* gene located on chromosome 20.	*In vitro* studies. CircITCH is an antitumor molecule, enhances *Itch* expression that further regulates the Wnt/β-Catenin pathway	TargetScan/TargetScanS, miRanda	Li F. et al., 2015
Cardiac fibrosis	CircHIPK3 (high)/miR-29b-3p (low)/a-SMA, COL1A1, COL3A1 (high)	Derived from exon 2 of *Hipk3* gene. Localized in cytoplasm	*In vitro* studies. miR-29b-3p has an antifibrotic effect and is a target for circHIPK3	regRNA2	Ni et al., 2019
Alzheimer's disease (AD)	CircHDAC9 (low)/miR-138 (high)/Sirt1 (low)	Localized in cytoplasm	*In vitro* studies CircHDAC9 has a binding site for miR-138 for which Sirt1 is a target. Sirt1 suppresses Amyloid-β production and regulates neuro-inflammation and mitochondrial dysfunction	TargetScan 7.0, miRanda 3.3a, RegRNA 2.0	Lu et al., 2019
	CiRS-7 (low)/miR-7 (high)/UBE2A (low)	CiRS-7 is found to have more than 70 miR-7 binding sites	UBE2A is an autophagic protein involved in amyloid peptide clearance in AD, and miR-7 is known to suppress its expression. CiRS-7 sponges miR-7 and declines its effect on UBE2A expression		Lukiw, 2013; Li et al., 2018

Similarly, a total of 115,171 circRNAs have been reported in 16 different plants (Chu et al., 2017), and 102 circRNAs have been found to contain miRNA binding sites in *S. lycopersicum* (Zuo et al., 2016). Pan et al. (2018) have used DEG software to trace out 20 circRNAs connected to the miRNA-mediated expression of 91 genes under heat shock conditions in *A. thaliana*. Nevertheless, a myriad of work demonstrating the circRNA sponging function in human diseases has been well documented as compared to plants.

Out of a significant number of circRNAs discovered so far in various organisms, only a handful of them have been validated, among which few are found to serve as miRNA sponges. In order to have effective sponging activity, the stoichiometry of the circRNA has to match with the abundance of the miRNAs. This could be attained through the presence of either multiple binding sites for a particular miRNA (CiR-7) or by having high copy numbers of the sponging circRNA, preferably with large-sized circRNA (Zheng et al., 2016). Small-sized circRNAs might have an inclination for exosomal-mediated ejection from the cell, which could be a reason for their inability to sponge (Li Y. et al., 2015; Zheng et al., 2016; Preußer et al., 2018). A single gene can generate multiple circRNAs, either with a high copy number, of different sizes (e.g., PTK2 gene can produce 47 distinct circRNAs) (Zheng et al., 2016), or in a combination of both possibilities. As already observed, a single circRNA can be expressed in various tissues (e.g., circHIPK3—Li et al., 2017; Ni et al., 2019) and can possess binding sites for multiple distinct miRNAs (e.g., circFOXO3—Han et al., 2017; Stefanetti et al., 2018). Considering all the cases mentioned above, a single circRNA can bind to multiple distinct miRNAs that regulate different pathways and *vice versa*, which is proof of a network that functions interdependently to maintain cellular homeostasis.

### CircRNAs as Potential Biomarkers and Therapeutic Targets

Discovering biomarkers at the early stages of a disease is a very promising path in diagnosis and prevention. While the race for detection and functional determination still continues for circRNAs, reports from many researchers are highlighting their potential as biomarkers and potential within therapeutics. Due to its high abundance, longevity, and tissue-specific expression, circRNAs could be a potential molecule used in forensic science. Reconstructing a crime scene is quite a challenging task due to limitations such as low quantity and quality of samples at the site. So far, RNA profiling is under investigation for potential to serve as a biomarker in the identification and differentiation of body fluids. Until the discovery of circRNAs, RNA-stable transcript regions and miRNAs have been studied as stable biomarkers, the latter being stable due to its small size and Argonaute binding. An effort was made to include circRNA in RNA profiling in order to enhance biomarker identification and sensitivity (Zhang Y. et al., 2018). It included circRNA of a peripheral blood-specific ALAS2 gene and a menstrual blood-specific MMP7 gene in RNA profiling to enhance identification and sensitivity. Further work is indeed needed to capitalize on its potential as a forensic biomarker. In addition to this, it has been shown that exosomes provide additional protection to circRNAs that are enclosed within it (Li Y. et al., 2015). Subsequently, an increasing amount of circRNA (up to ~2 fold) was found in exosomes when compared to the cells (Lu and Xu, 2016). Overexpressed small-sized circRNAs have a higher tendency to be expelled from cells as exosomes into the circulatory system, which can serve as a non-invasive diagnostics method for biomarkers. CircRNAs are found to be capable of crossing the blood–brain barrier (BBB), entering into the blood and cerebrospinal fluid (CSF) and can thus provide us with the status of Central Nervous System (CNS) disorders (Lu and Xu, 2016). On the other hand, the differential expression of plant circRNA profiles under a variety of stress conditions, such as drought in *T. aestivum* (Wang Y. et al., 2017), chilling in *S. lycopersicum* (Zuo et al., 2016), and nutrients stress, such as phosphate, iron, and zinc in *O. sativa* and *H. vulgare* (Darbani et al., 2016; Liu et al., 2017), might serve as reliable markers in plants that were previously underestimated.

### Cell to Cell Communication

About 1,215 circRNAs have been identified from isolated exosomes from human serum (Li Y. et al., 2015). Recently, the circulating exosomes containing overexpressed circRASSF2 were identified from laryngeal squamous cell carcinoma (LSCC) patients (Tian et al., 2019). These circulating exosomal circRNAs are usually found to be <1,000 nt long with a median of 350 nt in length (Li Y. et al., 2015). The small-sized circRNAs may become enclosed in exosomes and circulate in the blood to serve as potential biomarkers or as a cell signaling molecules. Moreover, the sorting of circRNAs in exosomes further depends on the low levels of its miRNA target(s) in the cells (Li Y. et al., 2015). Knowing their prolonged stability, the expulsion of extracellular vesicles (EVs) containing circRNAs is one of the evident ways for the clearance of circRNA cargo (Lasda and Parker, 2016). We speculate that the transport of these EV circRNAs might be involved in metastasis and proliferation of cancer. In contrast to animals, long-distance trafficking of PSTVd via the phloem (Palukaitis, 1987; Zhu et al., 2001) highlights the possibility of the circRNAs being communicating molecules via plant vasculature. Nevertheless, extensive research is indeed further required in this direction.

### Transcription Enhancer/Repressor

Recent discoveries have provided the functional aspect of circRNAs by exploring their potential as transcriptional regulators in both a *cis* and *trans* manner. Initially, the introns containing circRNAs, such as circEIF3J, circPAIP2, circANKRD52, circMCM5, and circSIRT7, are found to be interacting with the elongating RNA Polymerase II complex through positive feedback to regulate their own gene expression (Zhang et al., 2013). Similarly, nucleus-inhabiting EiciRNAs bind to U1 spliceosome components and promote expression of their own parental gene in addition to post-transcriptional regulation (Wilusz, 2017). Some circRNAs, such as circMBL, circFMN, and circDMD, can bind directly to their cognate mRNAs and thereby suppress their expression (mRNA trap) (Li et al., 2017). In plants, exon 6 *SEP3*-derived exonic circRNA tends to form an R-loop on the parental locus, thereby retarding its transcription elongation in *trans* to enhance the biogenesis of the exon-skipped circular variant (Conn et al., 2017). These studies demonstrate that the circRNAs can play an imperative role in diverse transcriptional regulation mechanisms.

### Cell Cycle Regulation

The diverse mechanism behind the action of the tumor suppressor gene-derived circFOXO3 in cell cycle regulation has been studied. Having binding sites for miR-22, miR-96, miR-136, miR-138, miR-149, miR-433, miR-762, miR-3614-5p, and miR-3622b-5p (Han et al., 2017; Stefanetti et al., 2018), circFOXO3 sponges these miRNAs from binding the linear variant of *FOXO3* and relieves its suppression. Besides having miRNA binding sites, circFOXO3 has binding sites for proteins involved in cell cycle regulation, such as p21, p27, p53, CDK-2, and MDM2. Two subsequent studies by Du et al. (2016, 2017) have emphasized the role of circRNA–protein interaction in cell cycle regulation using pull-down assays. They demonstrated the formation of a ternary complex by binding of p21 and CDK-2 to circFOXO3 at adjacent sites, which inhibits activation of the CDK-2/Cyclin-E complex otherwise necessary for G1/S transition, thereby resulting in cell cycle arrest in the G1 phase (Du et al., 2016). Along with this, circFOXO3 had binding sites for p53 and MDM2 and regulated the cell cycle (Du et al., 2017). To further strengthen its role in cancer development, low levels of circFOXO3 in breast cancer cell lines and patient samples were identified. Conversely, overexpression of circFOXO3 in cancer cells induced apoptosis and inhibited tumor growth (Du et al., 2017).

The role of circRNAs in the cell cycle regulation of cardiomyocytes has also been studied. *In situ* replenishment of cardiomyocytes after cardiac injury could be a potential recovery approach from damage incurred by a myocardial infarction (MI). Studies have demonstrated a reduced level of circNfix in proliferating neonatal cardiomyocytes when compared to adult cardiomyocytes (Huang et al., 2019). The ternary complex comprising of circNfix brings Nedd41 and YbX1 into close proximity and thereby mediates the ubiquitination and consequent degradation of YbX1. Downregulation of YbX1 leads to reduced levels of its downstream target genes, such as Cyclin A2 and Cyclin B1, and this ultimately inhibits cardiomyocyte proliferation. However, considerable research is needed to understand the mechanism in detail.

### Ribosomal RNA Maturation

CircRNA–protein interactions have further delineated the potential of circRNA to halt the global translational machinery in a cell besides commanding its own translation. circANRIL is one such example involved in the modulation of ribosomal RNA maturation, and it thereby dictates ribosomal biogenesis in the vascular smooth muscle cells and macrophages (Holdt et al., 2016). Using a lambda N peptide-mediated pull-down assay of circANRIL-B-Box, it was found that about 54% of the nuclear proteins were either involved in ribosomal biogenesis and its assembly or the regulation of rRNA splicing. A competitive attachment of circANRIL with a C-terminal lysine-rich domain of PES1 was also shown to prevent pre-rRNA binding. The latter resulted in dysfunction of the PeBoW complex, ultimately hindering the exonuclease-mediated rRNA maturation. Enhanced expression of circANRIL results in impaired ribosomal biogenesis due to premature rRNA accumulation as this increases p53 activation. This results in higher apoptosis and a lower rate of proliferation in humans, thereby manifesting the atheroprotective role of circANRIL (Holdt et al., 2016).

### Translation

CircRNAs are confidently grouped under long non-coding RNAs; a result of this is that the translational potential of circRNA has never been given much attention. The functional catalog of circRNAs was initially comprised of numerous evidence indicating miRNA sponging and protein sequestrating across animals and plants (Barrett and Salzman, 2016). However, recent evidence has highlighted the protein-coding potential of endogenous circRNAs due to their abundant association with the polysome (Legnini et al., 2017; Pamudurti et al., 2017; Yang et al., 2017). CircZNF609 from myoblasts presents in heavy polysomes and contains two in-frame start codons separated by 150 nt. It also carries a 5′ conserved internal ribosome entry site (IRES) and thus produces two similar intense proteins by cap-independent translation. Knockdown of circZNF609 using specific siRNA decreases proliferation of human and mouse myoblast cell lines, signifying its role in myogenesis (Legnini et al., 2017). Subsequently, it is investigated through an RNA-wide analysis on the m6A pattern that the IRES of circZNF609 is highly methylated (Zhao et al., 2014), which probably makes it responsible for the cap-independent translation. CircRNA translation requires the involvement of METTL3/METTL14, eukaryotic initiation factor eIF4G2, and m6A reader YTHDF3 compounded by heat stress. This leads to translocation of YTHDF2 from the cytosol to nucleus to block FTO, ultimately increasing m6A modification at its consensus motif (RRm6ACH, A–G/A and H–A/C/U) in IRES to initiate cap-independent translation (Yang et al., 2017). Therefore, replacement of circZNF609 IRES with a different UTR of same length inhibits its own translation (Legnini et al., 2017). However, a single m6A site is enough to induce circRNA translation with same efficiency as that by two m6A sites present in IRES (Yang et al., 2017). A summarized report from Yang et al. (2017) stated that 623 circRNAs were m6A methylated in the human Hs68 cell line, of which 25 circRNAs had a translation initiation site with ≥150 nt. On the other hand, 250 circRNAs were found to be associated with polysomes that correspond to ~0.6 circRNA/million reads with translatable coding potential (Yang et al., 2017). About 72 human circRNAs have been proven to express proteins as listed in circRNADb (Chen X. et al., 2016). Similarly, another endogenous circRNA from *Drosophila*, circMBL3, encodes ~37 KDa proteins and shows two specific bands (with and without the fifth exon). Besides, 34–158 circRNAs derived from *Drosophila*, rats, and mice were found to be associated with polysomes, emphasizing that those circRNAs might be translated into proteins (Pamudurti et al., 2017).

Protein translation from endogenous circRNAs informs the possible role of circRNAs in cancer progression. In an attempt, Zheng et al. (2019) found a ~10 KDa circPPP1R12A-73aa (coding 73 amino acids) protein expressed from circPPP1R12A (hsa_circ_0000423) that had a 216 nt short open reading frame formed by the backsplicing of exon 24 and 25. This circPPP1R12A-73aa protein has a unique conserved peptide GRLRHVNCLSPGVQD at the C-terminal. The circPPP1R12A-73aa protein, unlike the circPPP1R12A, regulates colon cancer progression, invasion, and metastasis, as was proven from 20 different patient samples and in nude mice. *In vitro* expression of the circPPP1R12A-73aa protein in various colon cancer cell lines, such as HT-29, HT-116, SW480, SW620, LoVo, SW48, DLD-1, CaCo2, and HCT-15, was also studied. The expression of the circPPP1R12A-73aa protein was found to increase in colon cancer cell lines as compared to control cell line NCM460 (Zheng et al., 2019). Similarly, a 17 KDa novel protein, SHPRH-146aa, was expressed from 440 nt circSHPRH (having an overlapping start and stop codon) after backsplicing of exons 26–29. It also possessed a unique peptide sequence, AAILQKWK, and is present more in normal brain tissue (Zhang et al., 2018a). PINT87aa, a 10 KDa protein expressed from circPINTexon2/circLINC-PINT (hsa_circ_082389), is formed by the circularization of exon 2 (Zhang et al., 2018b). Likewise, FBXW7-185aa, which is expressed from circFBXW7 (novel_circ_022705) after the backsplicing of exon 3 and 4, is a 21 KDa protein (Yang Y. et al., 2018). Both proteins are expressed more in normal brain tissue than in glioblastoma. PINT87aa may bind to the 150–300 aa domain of the PAF1 protein complex, which in turn recruits RNA polymerase II and regulates the transcriptional elongation of downstream genes (Zhang et al., 2018b). FBXW7-185aa promotes cell cycle arrest at the G1 stage and reduces the proliferation of glioma cells (Yang et al., 2017). Since circRNA may share the same coding sequence (CDS) as their corresponding linear mRNA, it is difficult to identify the origin of the translatable product. Moreover, library construction is difficult for ribosome footprinting (RFP) circRNAs due to the limited availability of tools for the identification of the circRNA-generated peptides. So far, no proteins or peptides have been detected from plant circRNAs. However, an elaborative study is required to search for m6A sites at 5′UTR in plants, and this could pave a path for the possibility of cap-independent translation.

## Role of CircRNA in Plants so Far

The search for the presence of circRNAs is ongoing in plants but at a slower pace than the ongoing search in animals. Though >100,000 circRNAs have been identified from different plants as listed in the plant circRNA database (Chu et al., 2017), only a fraction of those have been validated. The population of exonic circRNAs differs between plants and even species of the same plant, ranging from ~6.5 to 86% (Lu et al., 2015; Ye et al., 2015; Wang Z. et al., 2017; Zhao W. et al., 2017; Guria et al., 2019), due to usage of various pipelines, incomplete genome annotations, and other unknown possibilities. Different circRNAs are found to be expressed at different biotic and abiotic stress conditions, as has been proven in plants like *O. sativa* (Ye et al., 2015), *S. lycopersicum* (Zuo et al., 2016), *A. thaliana* (Pan et al., 2018), *T. aestivum* (Wang Y. et al., 2017), *P. betulifolia* (Wang et al., 2018), *A. deliciosa* (Wang Z. et al., 2017), and *S. tuberosum* (Zhou R. et al., 2017), where these circRNAs could act as potential plant biomarkers.

In the context of functional significance, plant circRNAs are found to have network interaction with miRNAs as sponging or cleavage properties (Chu et al., 2018b; Guria et al., 2019). However, this observation requires further validation either by the overexpression or knockdown of those circRNAs in plants. Although only a handful of plant circRNAs are having a putative miRNA interaction ability, its scope for other unidentified function cannot be sidelined, as was claimed in animal circRNAs. For example, exon 6-skipped circRNA of *SEP3* in *A. thaliana* forms a DNA–RNA hybrid loop that negatively regulates the transcription of its host gene (Conn et al., 2017). This reveals a novel function of circRNA that incites curiosity for the possibility of a similar mechanism in other plants as well. Similarly, circRNA derived from *PSY1* (involved in carotenoid biosynthesis) is found to be differentially expressed during various stages of fruit ripening (Tan et al., 2017). However, overexpression of PSY1-circ1 leads to decreased beta-carotene and lycopene content, resulting in yellowing of the fruits. Similarly, PDS-circ1 has also been shown to regulate the ripening pathway as a decrease in PDS mRNA expression often results in photobleaching of leaves, petals, and sepals (Tan et al., 2017). No plant circRNAs have so far been found to code for any proteins, although the presence of ORF in circRNA downstream of the IRES sequence complemented by m6A could act as a potential translatory endogenous circRNA. The biogenesis of plant circRNAs does not always follow the same pattern that is found in animals, such as having fewer repetitive or complementary flanking sequences (Zhao T. et al., 2017) and the presence of more non-GT/AG backsplice junctions (Guria et al., 2019). As a result, alternative circularization is frequent (Ye et al., 2017, Ye et al., 2015, Lu et al., 2015, Tan et al., 2017, Guria et al., 2019) because of which different types of circRNAs that originate from different loci are abundant in plants (Table 1). It is interesting to know that ~6 and ~1% of circRNA found in *A. thaliana* comes from chloroplast and mitochondrial genes, respectively, which is indicative of its presence and regulation in sub-cellular organelles (Sun et al., 2016). Trans-backsplicing is also significant in plants, with a reported 13% of circRNAs in *A. thaliana* and 34% in *O. sativa* (Ye et al., 2015; Chu et al., 2018b), apart from a single circRNA in *N. benthamiana* that was identified by the MDA-cloning method (Guria et al., 2019). However, the effect of these circRNAs on gene regulation and the possible physiological changes thereof need to be thoroughly evaluated. Most of the exonic circRNAs formed in *O. sativa, A. thaliana*, and *G. max* are comprised of 1–4 exons (Lu et al., 2015; Ye et al., 2015; Zhao W. et al., 2017), which are possibly formed post-transcriptionally after the intervening introns are removed; this is unlike single exonic circRNA, which is derived by the co-transcriptional pathway (Chu et al., 2018b).

There is an urgent need for the development of computational pipelines designed exclusively for plants as false positives often crop up during validation using animal- or human-specific software. Circseq_cup (Ye et al., 2017) has therefore been released to accurately explore the complete sequence of circRNA in plants, and it has ~3,000 assembled full-length circRNAs from *O. sativa*. PcircRNA_finder is the only plant-specific circRNA prediction software, although it uses multiple programs and yields only exonic circRNA (Chu et al., 2018a). Therefore, it is possible that the usage of various types of software enlists different types of circRNAs from the same plants, and this is probably due to discrete criteria for setting up the software. A vast plethora of circRNA-related research and the development of unique software are therefore required in plants as well as animals, which has immense potential in terms of unraveling various mechanisms that work together in plants.

## CircRNA Degradation

The fluctuating levels of circRNAs inside the cells upon stress stimulation (Ye et al., 2015; Liu P. et al., 2019) or during developmental differentiation have recently been studied (Mahmoudi and Cairns, 2019). Despite existing knowledge on circRNA degradation, more detailed testing is required to strengthen the lesser-known concepts. The regulation of circRNA degradation is known to be controlled by five pathways, some of which have been proven. What remains is hypothetical, which necessitates further validation.

The first pathway to mention is in accord to our previously published data (Guria et al., 2019), where we have computationally predicted a high percentage (~85%) of perfect complementary miRNA binding sites with plant circRNAs that may subsequently be subjected toward degradation. This is in line with previous findings that mention near-perfect complementarity between miRNA and its target in plants as compared to animals (Schwab et al., 2005; Ding et al., 2012). Although the interaction of circRNA and miRNA is broadly known through sponging models, the instance of miRNA-mediated regulation of circRNA degradation has so far barely been highlighted. It has been demonstrated in HEK293 cells that the sequence specificity between miR-671 and the non-linear natural antisense transcripts of *CDR1* directs the cleavage of the latter and regulates its mRNA levels (Hansen et al., 2011). It might be possible that many circRNAs are prone to degradation by Ago2-slicer-mediated action with its target miRNA as shown previously (Hansen et al., 2011); although further research is required to validate the above concept across eukaryotes.

Secondly, circRNA degradation could be carried out through endonuclease activity. The activation of cytoplasmic endonuclease RNaseL has recently been shown to trigger PKR activation via cleavage of bound inhibitory dsRNA in systemic lupus erythematosus patients (Liu C.-X et al., 2019). These patients further showed a reduced level of circRNAs, which were found to form a 16–26 bp dsRNA imperfect duplex. The latter, when complexed to PKR, inhibits its activity. Therefore, activation of RNaseL through the 2′ and 5′-oligoadenylate synthetase system upon viral stimulation targets even the dsRNA duplexes of circRNAs, along with the viral and cellular RNA, to activate the PKR via the antiviral signaling pathway (Liu C.-X et al., 2019).

Thirdly, the exosomes, due to their 3′-5′ exoribonuclease activity, are known to participate in mRNA quality control by being actively involving in mRNA processing and degradation. Besides this, it was recently found that, out of its nine subunits, the Rrp44 subunit possessed a PilT N-terminus (PIN)-domain with endoribonuclease activity. This may contribute to circRNA degradation, which is otherwise resistant to exonuclease activity due to its lack of linear ends (Schaeffer et al., 2009). Similarly, further detection of endo-ribonucleolytic activity of existing molecules and their interaction with circRNAs might open up the potential for a new mechanism for possible circRNA degradation.

The fourth pathway could be based on the known fact that m6A modified mRNAs that are recognized and guided by YTHDF2 into nuclear P-bodies for their degradation (Wang X. et al., 2014). Recently, it has been shown that 22% of 1,348 circRNAs interacting with YTHDF2 proteins are m6A modified in HeLa cells using RNA immunoprecipitation (RIP)-seq (Zhou C. et al., 2017). In addition, ciRS-7 has previously been found to compartmentalize in P-bodies when co-transfected with miR-7 in HEK293 and HeLa cells (Hansen et al., 2013). Therefore, it is highly possible that the interaction of YTHDF2 with m6A-modified circRNA may lead to its turnover. However, detailed investigation regarding its mechanism of action is indeed required to ascertain the proposed concept.

In addition to the above mentioned pathway, the possible fifth pathway could be EV mediated, in which EVs are membrane-bound structures capable of encapsulating cellular components, including circRNAs as mentioned previously (Yang and Li, 2018). Given their prolonged stability, the expulsion of EVs containing circRNAs is one of the ways the cell can get rid of the accumulating circRNA population (Lasda and Parker, 2016). It may become further degraded upon encountering endonucleases present in the extracellular matrix.

## Conclusion and Perspectives

Significant reports in the field of circRNA have illuminated the RNA world recently. Novel circRNAs are reported to have diverse functions; this includes acting as biomarkers and having therapeutic potential in uses for cancer and other diseases, which could further pave the way for effective diagnosis, treatment, and prevention. As compared to the animal system, research on plant circRNAs is minimal and is in need of more attention, especially designing of exclusive bioinformatic tools. Nevertheless, some of the proposed findings on plant circRNAs, including the mechanism of their biogenesis, which deviates from animal circRNAs, are worth mentioning; putative circRNA has, for example, a regulatory role in metabolic pathways. In the coming years, more research has to be put forward into the translational potential of circRNA. It is important to unravel any differences in the function of proteins coded by circRNA from the canonical spliced mRNA. It is equally interesting to study the possibility of protein isoforms generated from alternative backsplicing and their possible function in gene regulatory networks. It is also intriguing to know whether the secondary structure of circRNA will affect its miRNA sponging ability. Similarly, in the absence of a terminator codon in circRNA, how the protein synthesis is regulated is another interesting question to be resolved. Nevertheless, the hunt is on across the scientific community to find answers to many more burgeoning questions, including in the field of plant circRNAs.

## Author Contributions

AG and PS contributed equally in conceiving the review focus, conducting the literature review, summarizing the manuscript, reviewed literature, wrote the first draft, and finalized the manuscript. GP, SN, AG, and PS revised and made corrections to the manuscript. All authors approved the final version of manuscript.

### Conflict of Interest

The authors declare that the research was conducted in the absence of any commercial or financial relationships that could be construed as a potential conflict of interest.
